# Gilbert syndrome in Iran, Fars Province

**DOI:** 10.4103/0256-4947.59376

**Published:** 2010

**Authors:** Fariba Hemmati, Forugh Saki, Nasrin Saki, Mahmood Haghighat

**Affiliations:** a From the Department of Pediatrics, Shiraz University of Medical Sciences, Shiraz, Iran; b From the Department of Student Research Center, Shiraz University of Medical Sciences, Shiraz, Iran

**To the Editor:** Gilbert syndrome is a relatively common disorder of bilirubin metabolism that presents as unconjugated hyperbilirubinemia with otherwise normal liver structure and function.^1^,^2^ It needs no treatment or long-term medical attention. It is usually one of the differential diagnoses of liver or hemolytic disease, and could be mistaken as such based on liver function tests. The primary cause of this syndrome is decreased hepatic levels of glucoronosyl transferase. It occurs in about 3% to 7% of the population,^1^ but its prevalence differs by ethnicity. For example, in Saudis it was 3.6%,^3^ in Singapore 3.2%,^4^ and in Kashmir 3%.^5^ However, in one report from Germany, the prevalence was 12.4% in men and 4.8% in women.^6^ Reported ratios of male-to-female range from 1.5:1 to 7:1. Diagnosis of Gilbert syndrome is usually based on history, the findings of physical examination and some diagnostic tests such as phenobarbital, nicotinic acid, and rifampin tests, and specific genetic studies that are performed in special laboratories in some developed countries. Although genetic testing may be helpful for individual patients, the screening value of such genetic tests cannot be fully determined until accurate data are available for the prevalence and peneterance of the Gilbert syndrome genotype. Thus, genetic testing for Gilbert syndrome cannot be routinely recommended. To our knowledge, the prevalence of Gilbert syndrome has not yet been studied in the Iranian population. Hence, we designed this study to determine the prevalence rate in Iranian people of the Fars province.

We evaluated all persons who were referred for premarriage counseling or checkup to the Counseling Centre of Shiraz University from February 2007 to February 2009. Volunteers were included in our study after the details of our study were described to them. The history of blood and liver diseases were obtained from all of them and physical examination was done to rule out any stigmata of liver diseases. Complete blood and reticulocyte counts and liver function tests were performed for all the patients. Finally, a total of 460 persons whose history, physical examination, and laboratory tests revealed neither hemolytic nor liver disease, were taken as our subjects.

An overnight rifampin test was done for all patients wherein total serum and unconjugated bilirubin levels were measured from two blood samples. The first was taken before administering rifampin and the second, four hours after a single 600 mg oral dose of rifampin while the patient was fasting. Our criteria for the diagnosis of Gilbert syndrome were a total serum bilirubin level of more than 2.4 mg/dL and a serum unconjugated bilirubin level of more than 1.3mg/dL. These values have a high degree of sensitivity and specificity for the diagnosis of Gilbert syndrome according to the findings of previous studies.^1^,^7^

Our population comprised of 460 persons (227 male, 233 female), 88 persons of whom had Gilbert syndrome (58 male, 30 female) according to our criteria. The calculated prevalence of Gilbert syndrome was 19.1% in this population [Table 1]. The prevalence in male and females was 25.6% and 12.8%, respectively for a male-to-female ratio was 2:1. Based on our findings, we conclude that Gilbert syndrome is a common entity in Iran probably due to the higher number of consanguinous marriages in Iran, the method of screening (rifampin test), and the high genetic susceptibility in Iran.

**Table 1:** Number of people according to rifampin test results.

	Positive rifampin test	Negative rifampin test
Female	30	203
Male	58	169

**Total**	**88**	**372**
